# Delayed larval development in *Anopheles* mosquitoes deprived of *Asaia* bacterial symbionts

**DOI:** 10.1186/1471-2180-12-S1-S2

**Published:** 2012-01-18

**Authors:** Bessem Chouaia, Paolo Rossi, Sara Epis, Michela Mosca, Irene Ricci, Claudia Damiani, Ulisse Ulissi, Elena Crotti, Daniele Daffonchio, Claudio Bandi, Guido Favia

**Affiliations:** 1 Dipartimento di Patologia Animale, Igiene e Sanità Pubblica Veterinaria, Università degli Studi di Milano, Via Celoria 10, 20133 Milan, Italy; 2 Dipartimento di Scienze e Tecnologie Alimentari e Microbiologiche, Università degli Studi di Milano, Via Celoria 2, 20133 Milan, Italy; 3 Scuola di Bioscienze e Biotecnologie, Università degli Studi di Camerino, Via Gentile III da Varano 62032 Camerino, Italy

## Abstract

**Background:**

In recent years, acetic acid bacteria have been shown to be frequently associated with insects, but knowledge on their biological role in the arthropod host is limited. The discovery that acetic acid bacteria of the genus *Asaia* are a main component of the microbiota of *Anopheles stephensi* makes this mosquito a useful model for studies on this novel group of symbionts. Here we present experimental results that provide a first evidence for a beneficial role of *Asaia* in *An. stephensi*.

**Results:**

Larvae of *An. stephensi* at different stages were treated with rifampicin, an antibiotic effective on wild-type *Asaia* spp., and the effects on the larval development were evaluated. Larvae treated with the antibiotic showed a delay in the development and an asynchrony in the appearance of later instars. In larvae treated with rifampicin, but supplemented with a rifampicin-resistant mutant strain of *Asaia*, larval development was comparable to that of control larvae not exposed to the antibiotic. Analysis of the bacterial diversity of the three mosquito populations confirmed that the level of *Asaia* was strongly decreased in the antibiotic-treated larvae, since the symbiont was not detectable by PCR-DGGE (denaturing gradient gel electrophoresis), while *Asaia* was consistently found in insects supplemented with rifampicin plus the antibiotic-resistant mutant in the diet, and in those not exposed to the antibiotic.

**Conclusions:**

The results here reported indicate that *Asaia* symbionts play a beneficial role in the normal development of *An. stephensi* larvae.

## Background

Symbiotic bacteria are widespread in insects in which they play different roles, from providing nutrients, to affecting reproduction and speciation, among others [1]. Mosquitoes are vectors of a variety of infectious diseases that have a dramatic impact on public health, like malaria, yellow fever, dengue and chikungunya. Despite the common knowledge that these diseases are caused by microorganisms, the interactions between mosquitoes and their overall microbial community have not been deeply investigated. Acetic acid bacteria (AAB) are traditionally isolated from fermented foods and plant material [2,3]. In the last years, AABs have been described as emerging symbionts of insects being found associated especially with those with a sugar-feeding habit [4,5]. AAB of the genus *Asaia* have been shown to be stably associated with larvae and adults of the malaria mosquito vectors *An. stephensi*, *An. maculipennis* and *An. gambiae *[6,7] where they form a main component of the mosquito-associated microbiota. *Asaia* is a versatile symbiont being capable of cross-colonizing insects from phylogenetically distant taxa [8] and of vertical, venereal and paternal transmission [9].

However little is known about the effect of *Asaia* on the host. In *Drosophila melanogaster* AAB have been shown to regulate the microbiota homeostasis, by keeping under control pathogenic species following a fine-tuning of the host immune response [10,11]. In *An. gambiae*, it has been shown that *Asaia* titer in the host body is kept under control of the innate immune system and it massively proliferates in the hemolymph when the AgDscam component of the immune response is silenced [12]. *Asaia* spp. have been shown to fix nitrogen [13] and it might be suggested that the role of these symbionts is to provide the host insect with organic nitrogen, a capacity already proposed for gut symbionts in other insect models [14].

A frequently used strategy to investigate the effect of microbial symbionts on the host consists of their removal using antibiotic treatments to observe the effect on the host vitality and fitness [15,16]. A main limit of such a strategy is the lack of a suitable control, since the effects observed could be caused by direct effects of the antibiotic on the insect and/or on other components of the microbiota. Here we have adopted a different strategy, setting control experiments with *Asaia* resistant to the antibiotic treatment. By using this strategy we showed that *Asaia* contributes positively to the normal larval development of *An. stephensi*.

## Results and discussion

### *Asaia* is important for larval development

**Figure 1:**
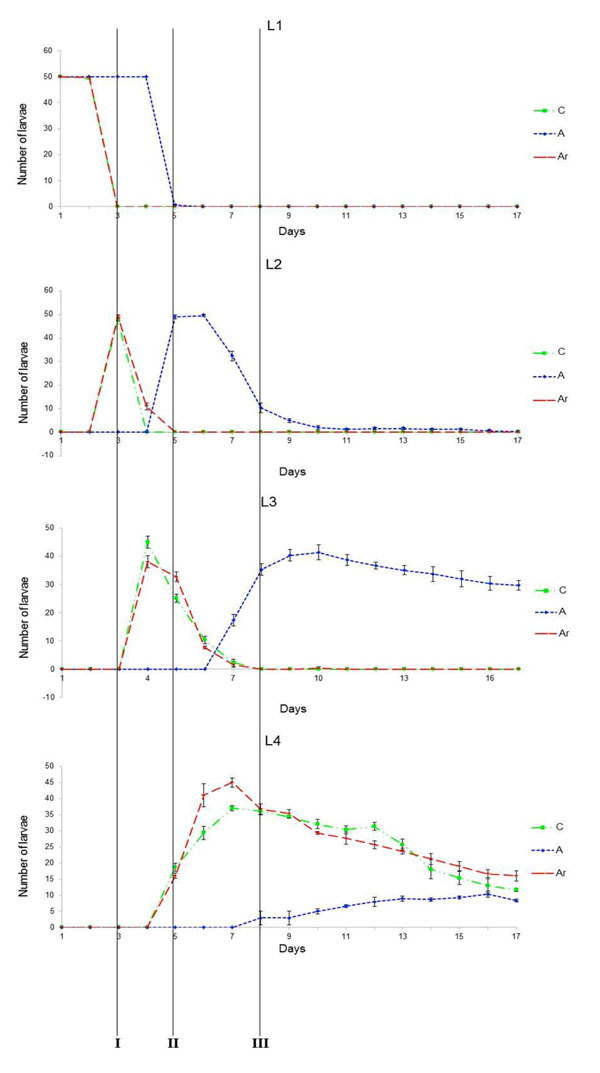
**Effects of rifampicin on mosquito larvae: developmental time is restored after administration of rifampicin-resistant *Asaia*.** Evolution of larval number at each different stage, in relation with time, when submitted to three different treatments. C: no treatment; A: rifampicin at 120 μg ml^-1^; Ar: rifampicin at 120 μg ml^-1^ plus rifampicin-resistant *Asaia*. L1: number of larvae at 1st instar; L2: number of larvae at 2nd instar. L3: number of larvae at 3rd instar; L4: number of larvae at 4th instar. I: time at which all the L1 non treated larvae molted to L2; II: time at which all the L2 non treated larvae molted to L3; III: time at which all the L3 non treated larvae molted to L4. Statistical analysis showed that the developmental rate of the larvae submitted only to the rifampicin treatment (A) is different from the two other cases (C and Ar; p < 0.05), for which the development time was not different. The X-axis reports the number of days and the Y-axis reports the number of the larvae at the stage indicated. In the case of the L1, the graph shows the disappearance of these larvae (i.e. their passage to the successive stage) from the starting number (50 for each experiment). In the other cases, the graphs report the appearance of the larvae at that stage, and then their disappearance (i.e. the passage to the successive stage).

**Figure 2:**
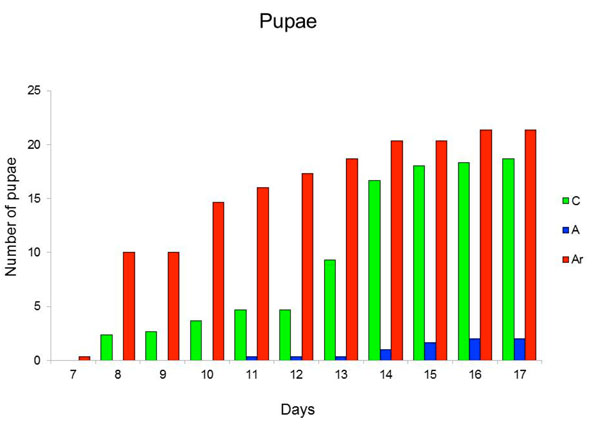
**Effects of rifampicin on larval development: the apparition rate of pupae is similar between non treated groups and rifampicin treated groups supplemented with a rifampicin-resistant Asaia.** The average cumulative number of pupae appearance, in relation with time, is reported for three different treatments. C: no treatment; A: rifampicin at 120 μg ml^-1^; Ar: rifampicin at 120 μg ml^-1^ plus rifampicin-resistant *Asaia*. The X-axis reports the number of days, starting from day seven, and the Y-axis reports the number of the pupae. The number of pupae at each day results from the sum of the pupae appeared at that day and the number of pupae counted in the days before.

The effects of rifampicin treatment on the *An. stephensi* larval development are reported in Figure 1 and 2. The developmental time of the larvae that were reared under rifampicin treatment (rearing batches A) was delayed 2-4 days depending on the larval stage, when compared to that of the control larvae (rearing batches C). The addition of a rifampicin- resistant *Asaia* to the breeding water (rearing batches Ar) restored the normal developmental time of the controls. Statistical analysis showed that the developmental time of larvae from groups (C) and (Ar) was significantly different from that of group (A) at all the developmental stages (respectively, Mann-Whitney U test, P=0.009 and Mann-Whitney U test, P=0.021).

The differences in the development time resulted in overall delays of the molting time, from two days at the 1st larval stage (Figure 1: L1) to more than four days in the 3^rd^ and 4^th^ stages (Figure 1: L3, L4). The graphs show that for the first two developmental stages (Figure 1: L1, L2) the larvae treated with the antibiotic follow a developmental curve similar to that of the control larvae (and of those supplemented with Ar in addition to the antibiotic), with the curve that is only shifted in time. For the latter developmental stages (Figure 1: L3, L4) the larvae treated with rifampicin showed very different curve shape. The appearance of the first larvae at these 3^rd^ and 4^th^ stages is also delayed in the group (A). In addition, we can also observe that in these stages (Figure 1: L3, L4) the larvae that are subjected only to the antibiotic treatment have a less synchronous appearance. This asynchronous development is not observed in treated larvae from previous stages (Figure 1: L1, L2). The loss of synchronicity appears when the larvae are passing from the L2 to the L3 stage. On the other hand, the control larvae and those treated with the antibiotic and supplemented with Ar remain synchronized in their development until the later L4 instar, and start to lose their synchrony only at the appearance of the pupal instar (Figure 1: L4; Figure 2).

Since dead larvae are almost impossible to spot into the water batches, particularly at the early stages, we were not able to directly determine the mortality in the different groups, although mortality could still be estimated indirectly, based on the number of the remaining larvae alive (considering also those removed throughout the study for molecular analysis). At the end of the experiment the cumulative number of living larvae in the different groups was similar, thus suggesting that removal of *Asaia* did not affect the mortality of the larvae. However, in the batches treated with antibiotic only (group A) a minor part of the larvae had molted to L4 when we interrupted the experiment (day 17; Figure 1: L3 and L4). In parallel, the number of pupae that developed in the group A was limited, compared to the pupae developed in groups C and Ar (Figure 2). Thus, even though the cumulative number of living larvae in the three groups was similar at the end of the experiment, in the group A more than half of the larvae were blocked at the L3 stage (Figure 1: L3).

### Larval developmental delay is concomitant with *Asaia* loss in the gut

**Figure 3:**
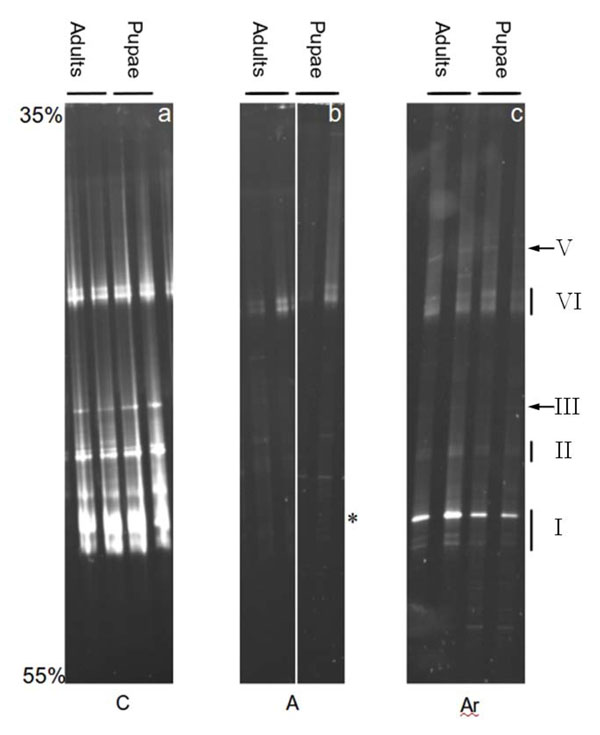
**Normal developmental time of mosquitoes is associated with amplification bands from *Asaia* in PCR-DGGE.** PCR-DGGE carried out on pupae and freshly molted adults of *An. stephensi* from the experimental groups of this study. a: DGGE on the non treated larvae. b: DGGE on the larvae treated with rifampicin at 120 μg ml^-1^. c: DGGE on larvae treated with rifampicin at 120 μg ml^-1^ and supplemented with rifampicin-resistant *Asaia*. I: bands at this level were identified as *Asaia* sp. after sequencing. II: bands at this level were identified as *Burkholderia* sp. III: bands at this level were identified as *Delftia* sp. Sequencing of bands at level VI was unsuccessful. V bands at this level were identified as *Anopheles* sp. 18S. * indicate the position in the gel form the larvae treated with antibiotics where *Asaia* bands were expected.

The larval microbiome tended toward a less heterogeneous community when the insect was fed with a rifampicin-based diet (Figure 3). Analysis of the bacterial diversity by PCR-DGGE (denaturing gradient gel electrophoresis) of 16S rRNA gene showed a remarkable simplification of the banding patterns, with the disappearance of several amplification products. In addition, besides the disappearance of most of 16S rRNA gene bands, the antibiotic treatment decreased the overall bacterial abundance, as shown by the low intensity of the bands remaining after the treatment in comparison with the control larvae (Figure 3). In the case of the larvae treated with antibiotic but supplemented with the rifampicin-resistant *Asaia* strain, the resulting bacterial community structure was simplified with respect to the untreated insects, while still showing bands that, after sequencing, were identified as coming from *Asaia bogorensis*. These bands co-migrated with a corresponding band in the control larvae that was also identified as *A. bogorensis* by band DNA sequencing (Figure 3). Other bands that have been sequenced are indicated in Figure 3a, and were identified as *Burkholderia* sp. and *Delftia* sp. Finally, quantitative PCR analysis on a subset of the samples showed that the levels of *Asaia* in the pupae were 1.2x10^7^, 1.4x10^2^ and 1.2x10^6^ in the C, A and Ar groups respectively. In adults, *Asaia* levels in the same groups were 4.9x10^7^, 6.8x10^2^ and 1.1x10^6^. These quantitative PCR results indicate that the antibiotic actually decreased the Asaia level, and that the Asaia load was restored when antibiotic-resistant bacteria were added into the cages.

It has already been shown that antibiotic treatment can strongly affect the structure of the bacterial community of insects. For instance, Lehman et al. [17] observed a modification in the microbial community associated with the predatory ground beetle (*Poecilus chalcites*) when transferred from the environment to a rearing facility. This modification was greater after antibiotic treatments, and was characterized by a loss of heterogeneity of the microbiota. However the microbial community was not completely eliminated.

In the case of *An. stephensi* larvae, rifampicin treatment determined a profound modification of the microbiome that was evidenced by a loss of bands in the PCR-DGGE profiles and a remarkable decrease of intensity as well. DGGE banding patterns indicated that the insects displaying a delayed development were actually deprived of *Asaia* presence. One could argue that the first effect (disappearance of *Asaia*) is not the cause the second one (delayed developmental time). However, when a rifampicin-resistant *Asaia* strain was supplemented to the diet, the normal larval development was restored. In addition, PCR-DGGE analysis revealed a sole difference between treated larvae (with delayed development), and treated larvae supplemented with the antibiotic-resistant *Asaia* (with normal development), i.e. the reappearance of the *Asaia* bands. In summary, our experiments provide evidence that *Asaia* plays a beneficial function for the normal mosquito larval development.

The fact that *Asaia* is the major inhabitant of the gut in *An. stephensi *[7], and that it is transmitted to the progeny by different ways [7][9], is also in agreement with the idea that this alpha-proteobacterium has a beneficial role for the insect. Even though we did not generate experimental evidence that could indicate the specific function for *Asaia*, some hypothesis can be proposed. The negative effects of *Asaia* loss on the larval growth of *An. stephensi* increase with the advancement of the development, in parallel with the increased metabolic requirement. We could thus suggest that *Asaia* is involved in the supply of nutrients to the host, like a nitrogen source [13], or vitamins, or other essential nutritional factors. But this does not exclude the possibility that *Asaia* can play a role in the development/homeostasis of the immune system of the host, as shown for other acetic acid bacteria that contribute to the proper functioning of the host insect immunity [11].

## Conclusions

Antibiotic removal of bacterial symbionts is a classic experimental strategy in studies on invertebrate symbioses. After administration of an antibiotic to the host, which is supposed to be effective on a given symbiont, physiological/pathological effects on the host are recorded, with the goal of getting clues on the biological role of the symbiont under study [15]. This strategy is however flawed by the multiple effects associated with antibiotic treatments, from direct effects on the host, to effects on other components of the microbiota. Here we have adopted a novel strategy, consisting in the administration antibiotic-resistant symbionts to antibiotic-treated individuals. In our study, the simple observation of a delay in the development in *An. stephensi* larvae after rifampicin treatment, in parallel with a dramatic reduction of *Asaia* burden, led to the hypothesis that this bacterium plays a beneficial role in the development of the mosquitoes. The restoration of the normal developmental time after administration of rifampicin-resistant *Asaia* provides a strong support to the above hypothesis. However, our work does not prove that *Asaia* is necessary for mosquito development. Indeed, we cannot exclude that a normal developmental time could be restored after administration of other microorganisms. On the other side, it is clear that introduction of antibiotic-resistant *Asaia* is sufficient for restoring mosquito development. In summary, while our results indicate that *Asaia* is sufficient for allowing a normal mosquito development, we cannot conclude that this bacterium is necessary, since we have not tested the administration of other bacteria. It is worth to remark that bacteria of the genus *Asaia* are found in the environment [18], and typing and phylogenetic studies did not reveal a specific clustering of strains collected from insects, as compared with environmental strains [19]. In addition, *Asaia* can be transmitted horizontally not only among insects of the same species [9], but also cross-colonizing insects from phylogenetically distant orders [4]. Finally, individual mosquitoes have been detected to host more than one strain of *Asaia *[19]. Overall, the results of our current work, and those of previous studies, do not argue for *Asaia* as an obligatory mutualist of *An. stephensi*, but as secondary, non essential, but beneficial symbiont of this insect.

## Material and methods

### Strains and rearing conditions

The experimental work was performed using a colony of *An. stephensi* (Liston strain) reared in the insectary of the Laboratory of Parasitology (University of Camerino, Italy) since 1988. The larvae were kept in 300 ml-volume transparent plastic containers, with a light period of 12:12 (Light:Dark) and a room temperature at 30°C. Larvae were fed with sterile minced commercial mouse food: Mice standard diet G.L.P. (Mucedola s.r.l. Italy)

### Antibiotic stability test

A test was carried out to check the stability of the antibiotic under the experimental conditions. The antibiotic (rifampicin) was put in a solution of water and food (concentrated at 0,4 g l^-1^) at a concentration of 120 μg ml^-1^ and left for 30 days at the rearing condition mentioned above. Every two days the efficiency of the antibiotic was tested with well-diffusion method [20] on a fresh culture of strain SF2.1 *Asaia*., isolated from *An. stephensi* [10; thereafter *Asaia* SF2.1].

### Generation of a rifampicin-resistant *Asaia* SF2.1 spontaneous mutant

*Asaia* SF2.1 was cultivated in GLY liquid medium (2.5% glycerol and 1% yeast extract, pH 5) until they reached OD_600_ of 1 (equivalent to 10^8^ CFU per ml), and 100 μl of the culture were plated on solid GLY medium (2.5% glycerol and 1% yeast extract, 20% agar, pH 5) supplemented with 100 μg ml^-1^ of rifampicin to obtain a spontaneous rifampicin-resistant mutant. After 96h of incubation at 30°C, one rifampicin-resistant colony, out of the 10 colonies obtained, was selected and transferred on liquid GLY medium and incubated until OD_600_ of 1. Then the cells were centrifuged and the pellet was conserved at 4°C to be used later.

### Function investigation

After assessing that rifampicin was stable and active for 30 days in larval rearing conditions (see antibiotic stability test), we started the experimental work on the larvae. The investigation of the possible role of *Asaia* was carried out monitoring three study cases: (i) larvae in water + food, i.e. the control case (C); (ii) larvae in water +food + antibiotic (A) at a concentration of 120 μg ml^-1^; and (iii) larvae in water+food+antibiotic+rifampicin-resistant *Asaia* (Ar). Each study case was conducted in triplicate. The antibiotic used was rifampicin, an mRNA synthesis inhibitor. In each case 50 larvae were used in 300 ml of a previously autoclaved medium (water plus food at the concentration of 0,4 g l^-1^). Each day a count was realized. The monitoring of the experiment was carried for 18 days. When reaching the pupal stage, half of the pupae were sampled and conserved for further analysis. The second half of the pupae was let to molt; after emergence, adults were immediately sampled and conserved.

### Statistical analysis

The results were analyzed to assess if there is a statistically significant difference between the treated larvae and the controls, in terms of mortality and development. The statistical analyses were carried out using the non parametric test U of Mann-Whitney under the SPSS software (ver.17, SPSS inc, USA). Values of P < 0.05 were considered as statistically significant.

### Analysis of the bacterial community of *An. stephensi*

Total DNA was extracted from pupae and adults of *An. stephensi* using the CTAB method with a prior cell lysis by enzymatic method and followed by an isopropanol precipitation of the DNA, as described by Jara et al. [21]. PCR amplification for DGGE was carried out using primers 357f (5'-CCTACGGGAGGCAGCAG-3') and 907r (5'-CCGTCAATTCCTTTRAGTTT-3') with a GC clamp, as described by Sanchez et al. [22].

DGGE (Denaturant Gradient Gel Electrophoresis) analysis was carried out on each PCR amplicon using a DCodeTM Universal Mutation Detection System (BioRad, Hercules, USA), following the procedure described previously [23]. Electrophoresis was performed in 0.5-mm polyacrylamide gel (7% (w/v) acrylamide–bisacrylamide 37.5:1) containing a 35–55% urea–formamide denaturing gradient (100% corresponds to 7M urea and 40% (v/v) formamide) according to the method of Muyzer et al. [24], increasing in the electrophoretic run direction. The gel was subjected to a constant voltage of 90 V for 15 h at 60 °C in TAE Buffer 1X (50X TAE stock solution consisting in 2 M Tris base, 1 M glacial acetic acid, 50 mM EDTA). After electrophoresis, the DGGE gels were stained in 1X TAE solution containing SYBR Green (Molecular Probes, Leiden, The Netherlands) for 45 min and photographed under a UV illumination using a GelDoc 2000 apparatus (BioRad, Hercules, USA). For the sequencing of DGGE bands, bands of interest were excised from the gels with a sterile blade, mixed with 50 μl of sterile water, and incubated overnight at 4°C to allow the DNA of the bands to diffuse out of the polyacrylamide gel blocks. Two microliters of this aqueous solution was used to reamplify the PCR products with the same primers described above, excluding the GC clamp. Reamplified bands were then sequenced using ABI technology [22].

## Quantitative Real-Time PCR

Quantitative PCR (qPCR) was performed, on a subset of 18 samples (nine adults and nine pupae) grouped in pools of three taken from each cage, with a IQ5-cycler thermal cycler (Bio-Rad) using *Asaia* specific primers Asafor (5'-GCGCGTAGGCGGTTTACAC-3') and Asarev (5'-AGCGTCAGTAATGAGCCAGGTT-3'), 0.3 µM each. The concentration of each insect DNA sample was measured with a Nanodrop ND-1000 spectrophotometer, and 5 ng DNA was used in 25-µl reactions. For Asaia qPCR an initial denaturation at 94°C for 3 min was followed by 40 cycles consisting of denaturation at 94°C for 30 sec, annealing at 60°C for 30 sec. For both the qPCR a final step for melting curve analysis from 70 to 95°C, measuring fluorescence every 0.5°C, was added. PCR products for standard curve were cloned using pGEM T-easy Vector Cloning Kit (Promega). Standard curves had an average correlation coefficient of 0.998, a slope of -3.663, with a PCR efficiency of 95% for Asaia specific qPCR.

## List of abbreviations used

(C): control cages for mosquito larvae (water + food); (A): cages for mosquito larvae, with administration antibiotic (water + food + antibiotic); (Ar): cages for mosquito larvae, with administration antibiotic and antibiotic-resistant *Asaia* (water + food + antibiotic + antibiotic-resistant *Asaia*) [for further explanation on (C), (A), and (Ar), see Material and Methods and Results and Discussion]; DGGE: denaturing gradient gel electrophoresis; AAB: acetic acid bacteria; L1, L2, L3, L4: mosquito larvae at the first, second, third and fourth stage.

## Author’s contributions

BC, SE, PR, CD, UU, MM and IR designed and performed most of the experiments and analyzed data EC and DD contributed to data analysis and writing the paper, CB and GF conceived the research, designed and supervised all the experiments and wrote the paper. All authors have read and approved the final manuscript.

## Competing interests

The authors declare that they have no competing interests.
